# Effect of Age on Clinical Trial Outcome in Participants with Probable Alzheimer’s Disease

**DOI:** 10.3233/JAD-210530

**Published:** 2021-08-03

**Authors:** Steven D. Targum, Lisa Fosdick, Kristen E. Drake, Paul B. Rosenberg, Anna D. Burke, David A. Wolk, Kelly D. Foote, Wael F. Asaad, Marwan Sabbagh, Gwenn S. Smith, Andres M. Lozano, Constantine G. Lyketsos

**Affiliations:** a Functional Neuromodulation Ltd., Minneapolis MN, USA; b Memory and Alzheimer’s Treatment Center & Alzheimer’s Disease Research Center, Division of Geriatric Psychiatry and Neuropsychiatry, Department of Psychiatry and Behavioral Sciences, Johns Hopkins University School of Medicine, Baltimore, MD, USA; c Department of Neurology, Barrow Neurological Institute, Phoenix, AZ, USA; d Penn Memory Center, Department of Neurology, University of Pennsylvania, Philadelphia, PA, USA; e Departments of and Neurosurgery and Neurology, University of Florida, Fixel Institute for Neurological Diseases, Gainesville, FL, USA; f Department of Neurosurgery, Rhode Island Hospital and the Alpert Medical School of Brown University, Providence, RI, USA; g Cleveland Clinic Lou Ruvo Center for Brain Health, Cleveland, OH, USA; h Department of Surgery (Neurosurgery), University of Toronto, Toronto, ON, Canada

**Keywords:** Age, Alzheimer’s disease, deep brain stimulation, clinical trials, subject selection

## Abstract

**Background:**

Age may affect treatment outcome in trials of mild probable Alzheimer’s disease (AD).

**Objective:**

We examined age as a moderator of outcome in an exploratory study of deep brain stimulation targeting the fornix (DBS-f) region in participants with AD.

**Methods:**

Forty-two participants were implanted with DBS electrodes and randomized to double-blind DBS-f stimulation (“on”) or sham DBS-f (“off”) for 12 months.

**Results:**

The intervention was safe and well tolerated. However, the selected clinical measures did not differentiate between the “on” and “off” groups in the intent to treat (ITT) population. There was a significant age by time interaction with the Alzheimer’s Disease Assessment Scale; ADAS-cog-13 (*p* = 0.028). Six of the 12 enrolled participants < 65 years old (50%) markedly declined on the ADAS-cog-13 versus only 6.7%of the 30 participants≥65 years old regardless of treatment assignment (*p* = 0.005). While not significant, *post-hoc* analyses favored DBS-f “off” versus “on” over 12 months in the < 65 age group but favored DBS-f “on” versus “off” in the≥65 age group on all clinical metrics. On the integrated Alzheimer’s Disease rating scale (iADRS), the effect size contrasting DBS-f “on” versus “off” changed from +0.2 (favoring “off”) in the < 65 group to –0.52 (favoring “on”) in the≥65 age group.

**Conclusion:**

The findings highlight issues with subject selection in clinical trials for AD. Faster disease progression in younger AD participants with different AD sub-types may influence the results. Biomarker confirmation and genotyping to differentiate AD subtypes is important for future clinical trials.

## INTRODUCTION

Age of onset can be a confounding moderating variable in clinical trials of probable Alzheimer’s disease (AD) because many younger, early onset AD participants have a more rapid cognitive decline than later onset participants that may be associated with distinct genetic subtypes [1–5]. Using data obtained from 10 studies and 2,793 probable AD participants, Schneider and colleagues [4] reported that the younger participants had faster rates of cognitive decline than the older participants on the Alzheimer’s Disease Assessment Scale (ADAS-cog) over 12–24 months (*p* < 0.0001).

In a recent study, we found that age affected the clinical outcome in study participants with probable mild AD treated with deep brain stimulation targeting the fornix (DBS-f) as the experimental condition [6]. The rationale for using DBS in AD is based upon the hypothesis that modulation of neuronal circuits may sustain or possibly even improve memory function in affected individuals [7–9]. Many investigators have described functional alterations in the interconnected cortical networks associated with memory in AD participants, particularly in areas related to the hippocampus [10, 11]. The rationale to focus DBS on patients with early illness rather than moderate AD was supported by a pilot study that showed greater benefits from DBS in probable AD participants who were less cognitively impaired and showed less severe cerebral glucose metabolic deficits prior to the intervention [7, 12, 13]. Given this rationale, a broad age range of potential study participants (45–80) who met criteria for probable mild AD were considered eligible for this Phase 2 exploratory study. The ADAS-cog-13 and Clinical Dementia Rating Scale (CDR) results failed to differentiate between AD participants assigned to DBS-f “on” (stimulation) or “off” (sham) after 12 months of blinded treatment although the FDG-PET imaging findings revealed that DBS-f “on” stimulation increased metabolic function relative to the sham treatment group [6, 14, 15]. There was an unusually high percentage of younger probable AD participants in this small study that may have impeded signal detection. Although less than 4%of the AD population are reported to develop the disease before the age of 65, 12 of the 42 enrolled participants (28.6%) in this study were < 65 years old [6, 16, 17]. A *post-hoc* multivariate regression analysis revealed a significant age interaction with time and experimental condition on the ADAS-cog-13 (β= –0.41; SE 0.18; *p* = 0.028). Participants < 65 years old had greater cognitive decline and lower pre-treatment glucose metabolism in temporal and parietal cortices than the older participants regardless of treatment assignment [6].

In our initial reports of this study, we described the safety and tolerability of the intervention as well as the ADAS-cog and CDR results [6, 18, 19]. In this *post-hoc* analysis, we report additional clinical results from two secondary outcome measures, the ADCS-Activities of Daily Living scale (ADCS-ADL-23) and the integrated Alzheimer’s Disease Rating Scale (iADRS) [20–23]. The iADRS provides a composite score that combines the cognitive measures of the ADAS-cog-13 with the instrumental items of the ADCS-ADL-23 [14, 20, 21]. In two recent clinical trials, the iADRS was chosen as the primary efficacy measure and was able to demonstrate a statistically significant slowing of clinical decline favoring solanezumab over placebo in one study, and donanemab over placebo in the other study [22, 23]. Our *post-hoc* analysis of the iADRS in the ADvance study confirms that age was a pivotal moderating factor affecting signal detection and offers data that may be useful for the design of future AD trials.

## MATERIALS AND METHODS

Data for this report were derived from the phase II AD study *ADvance: A Twelve Month Double-blind Randomized Controlled Feasibility Study to Evaluate the Safety, Efficacy and Tolerability of Deep Brain Stimulation of the Fornix (DBS-f) in Participants with Mild Probable Alzheimer’s Disease* sponsored by Functional Neuromodulation LLC. The trial was overseen by the Food and Drug Administration (USA) and by Health Canada and registered with clinicaltrials.gov (NCT01608061). Seven clinical trial sites located in the United States and Canada participated in the study that was conducted between May 2012 and June 2015 (last patient enrolled in March 2014). The study was conducted in compliance with local Institutional Review Board (IRB) informed consent regulations and International Conference on Harmonization (ICH) for Good Clinical Practice (GCP) Guidelines.

All potentially eligible participants personally provided informed consent and signed an IRB approved consent form at the screening visit with input from identified caregiver/informants. Participants and caregivers (informants) signed a second consent form at the baseline visit prior to DBS-f implantation.

### Study eligibility criteria

Eligible participants were men or women between the ages of 45 and 80 years living at home with a reliable informant, who had a General Medical Health Rating≥3 (good or excellent), and who met criteria for probable AD according to the National Institute of Aging/Alzheimer’s Association criteria [24]. An essential component of eligibility was a narrative documentation of memory complaints affecting behavior or daily function for at least a year, evidence of functional decline over the past year, and a stable dose of a cholinesterase inhibitor (donepezil, galantamine, or rivastigmine) for at least 60 days prior to signing the informed consent. In addition, eligible participants had a Clinical Dementia Rating Scale (CDR) global rating of 0.5 or 1.0, and an Alzheimer’s Disease Assessment Scale-cognitive component (ADAS-cog-11) score of 12–24 (inclusive) with a score≥4 on ADAS-cog item 1 (immediate recall) at both the screening and baseline visits [14, 15, 25].

The exclusion criteria included scores of≥11 on the Young Mania Rating Scale, > 10 on the Cornell Scale for Depression and Dementia, >4 on the modified Hachinski ischemia score, and≥10 on the Neuropsychiatric Inventory (NPI) total score (or≥4 in any domain except apathy) at the screening visit [14, 15, 25–28]. Potential participants were excluded if they replied “yes” to “suicidal ideation” or “yes” to any items in the suicidal behavior section with reference to the three-month period prior to screening on the Columbia Suicide Severity Rating Scale (C-SSRS) or had attempted suicide within the past 2 years [29]. Participants had to be considered a good surgical candidate by the neurosurgeon, free of contraindications for surgery or exclusions for magnetic resonance (MR) imaging (pacemakers, metal implanted in the body) or positron emission tomography (PET) scanning (e.g., insulin-dependent diabetes). Detailed inclusion/exclusion criteria for the ADvance study have been previously published [30].

The study protocol did not require *APOE* or CSF biomarkers as part of the study eligibility criteria. The subject eligibility process included pre-screening by both the trial site and a 3-member site-independent enrollment review committee (ERC) composed of psychiatrists, neurologists, and neurosurgeons who examined eligibility documentation for every potential study participant. The ERC reviewed all documentation related to participant eligibility including the documentation of functional decline in the past year. An approval to proceed toward randomization and implantation required the unanimous decision of the ERC.

### Study design

**Fig. 1:**
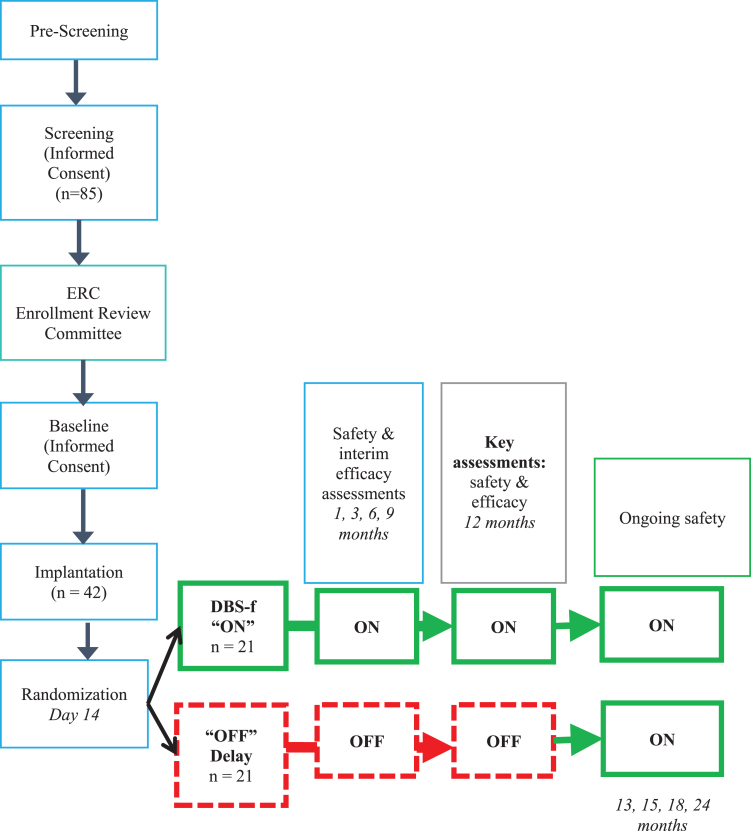
Schematic of Advance AD study design (CONSORT).

Figure 1 provides a schematic outline of the ADvance design. The 12-month DBS-f “on” (stimulation) or “off” (sham treatment) 1:1 randomization period following implantation was followed by a 12-month open-label extension for all participants. A more detailed description of the study methods, including the neuroimaging methods and the standardization of the surgical procedures have been published elsewhere [6, 18, 30].

A CRO (clinical research organization) statistician generated the 1:1 random allocation sequence that was stratified by the site using SAS according to the CRO’s standard operating procedures. Only the statistician knew the block sizes. A sponsor clinical engineer and an unblinded site technician carried out the randomization and recorded the blinded code number at the site. All other trial site staff, study participants, families, CRO, and sponsor personnel were blinded to the randomization allocation throughout the study.

### DBS neurosurgical implantation

The target site for neurosurgical implantation of the DBS electrodes for AD was just anterior and in juxtaposition to the post commissural fornix (DBS-f). The fornix, a critical part of the Papez circuit, was chosen for DBS in mild AD because it is a major inflow and output pathway from the hippocampus and medial temporal lobe [7, 18]. Furthermore, there is accumulating evidence from pre-clinical and human imaging studies suggesting that the fornix is involved early in cognitive decline [8, 9]. A Model 37601 Activa PC pulse generator battery and Model 3387 Leads with Model 37085 extensions (supplied by Medtronic, Inc.) was implanted in all participants who continued to meet randomization criteria at the baseline visit. A more detailed description of the neurosurgical procedure and safety outcomes are provided elsewhere [7, 18].

### Neuroimaging

All eligible participants underwent 1.5TMR scans at baseline and 12 months to obtain a structural image and [^18^F]-2-deoxy-2-fluoro-D-glucose PET (FDG-PET) at baseline and at 1, 6, and 12 months after surgery with the stimulators maintained ‘on’ in the active group and ‘off’ in the sham group during the PET scans [6].

### Clinical outcome measures

ADvance was a feasibility study in which the primary objective was to evaluate the safety and tolerability of DBS-f in participants with probable mild AD by assessing device and/or therapy related adverse events after 12 months. The secondary objective was to provide a preliminary estimate of the treatment effect size on selected clinical psychometric measures and neuroimaging outcomes at 12 months post-randomization.

The primary outcomes were the ADAS-Cog-13 and CDR sum of boxes (CDR-SB) at 12 months [14, 15, 25]. The Alzheimer’s Disease Cooperative Study Activities of Daily Living scale (ACDS-ADL-23) scale was a secondary outcome [20]. We added the integrated Alzheimer’s Disease Rating Scale (iADRS) in this *post-hoc* analysis [21, 22]. The iADRS is a composite of the ADAS-cog-13 and instrumental items of the ADCS-ADL (iADL) scores.

### Statistical analyses

The study objectives did not include formal tests or hypotheses as it was not powered to detect a statistically significant difference between treatment arms. All analyses were initially performed on the intention-to-treat (ITT) population that represented all participants who were randomized. In a *post-hoc* analysis, the ITT population was divided into two age cohorts made up of participants < 65 years old and≥65 years old. Descriptive statistics compared treatment groups on baseline variables. Between-group comparisons for change from baseline were made using ANOVA or *t*-tests and 2-sided *p*-values at each time point. Cohen’s d effect size (ES) was calculated as the expected difference in change at 12 months divided by the pooled standard deviation [31]. All analyses were performed with SAS software, version 9.3 or greater.

## RESULTS

Adherence with study procedures was excellent throughout the study. It was possible to obtain both baseline and 12-month (endpoint) ADAS-cog-13 scores on all 42 participants and CDR-SB and ADCS-ADL-23 scores on 41 participants. The primary measures (ADAS-cog-13 and CDR-SB) did not differentiate between the DBS-f “on” and “off” groups after 12 months of double-blind treatment. A key *post-hoc* finding was that participants < 65 years old had a more rapid cognitive decline and lower glucose metabolism in temporal and parietal cortices than the older participants regardless of treatment assignment [6].

### Demographic and baseline data

Participants ranged in age from 48 to 80 years old. Twelve participants were < 65 years of age (28.6%of the study population). All randomized participants had a documented history of behavioral and/or daily functional difficulties attributed to memory complaints for at least one year and had been taking cholinesterase inhibitor medication for at least 60 days prior to signing the informed consent. All participants were diagnosed with mild probable AD and had global CDR scores of 0.5 or 1.0. The mean time since the formal diagnosis of probable AD had been made by a physician was approximately two years, although some participants had been diagnosed nearly 6 years before the start of the study. The total ADAS-cog-11 scores for the 42 randomized participants at the baseline visit ranged from 12 to 22 (mean = 16.9±2.9 SD).

Table 1 lists demographic characteristics and baseline clinical scores for the 42 randomized participants stratified by age (< 65 years old versus≥65 years old).

**Table 1:** Characteristics of Randomized Participants in the ADvance DBS-f Study of Mild AD (stratified by age cohorts)

Patient Characteristics	Age < 65	Age≥65
*n*	12	30
Male gender	5 (42%)	18 (60%)
Age (y)		
Mean±SD	57.7±4.9	72.4±3.7
[Median] (min, max)	[58.6] (48.0, 64.8)	[72.4] (66.2, 79.7)
Time since diagnosis (y)		
Mean±SD	2.1±1.7	2.4±1.8
[Median] (min, max)	[1.5] (0.3, 5.9)	[2.0] (0.0, 5.9)
Level of education		
High school	0 (0%)	11 (37%)
College	7 (58%)	8 (27%)
Graduate school	5 (42%)	11 (37%)
ADAS-cog-13 at baseline		
Mean±SD	27.0±4.2	28.2±3.8
[Median] (min, max)	[27.5] (20.0, 34.0)	[28.0] (22.0, 36.0)
CDR Global score at baseline		
0.5	5 (42%)	23 (77%)
1	7 (58%)	7 (23%)
Mean±SD	0.8±0.3	0.6±0.2
CDR Sum of Boxes (CDR-SB) at baseline		
Mean±SD	4.3±1.5	3.6±1.4
Activities of Daily Living (ADCS-ADL-23) at baseline		
Mean±SD	69.9±4.8	69.3±6.5
Integrated Alzheimer’s Disease Rating Scale (iADRS) at baseline		
Mean±SD	91.2±7.2	89.3±8.5

Twenty-eight participants (67%) had CDR global scores of 0.5 and 14 participants (33%) had CDR global scores of 1.0 at the baseline visit. The mean CDR-SB score was 3.9±1.7 with a wide score range from 1.0 to 8.0 at the baseline visit. There were no significant differences between the < 65 years old and≥65 years old participants on baseline mean ADAS, CDR, ADCS-ADL, or iADRS. The ADAS-cog-13 scores were correlated with the CDR-SB scores at baseline (*r* = 0.459) and at 12 months (*r* = 0.676). Similarly, the iADRS scores were highly correlated with the CDR-SB score at baseline (*r* = –0.735) and at 12 months (*r* = –0.859) and the correlation did not differ between the age cohorts.

### Clinical outcomes from the ITT population

The study’s primary report was published elsewhere and can be summarized as follows: the mean change scores of the primary outcomes (ADAS-cog-13 and CDR-SB) revealed progressive cognitive decline over 12 months consistent with mild AD *but* did not distinguish between the DBS-f “on” and “off” treatment groups in the ITT population [6].

### Post-hoc analyses

As previously reported [6], a *post-hoc* multivariate regression analysis of the ITT population revealed a significant time by age interaction with ADAS-cog-13 outcomes (beta = –0.41; SE 0.18; *p* = 0.028): the 12 participants < 65 years old had greater cognitive decline and decreased glucose metabolism over 12 months regardless of treatment assignment than the 30 older participants [6].

**Fig. 2:**
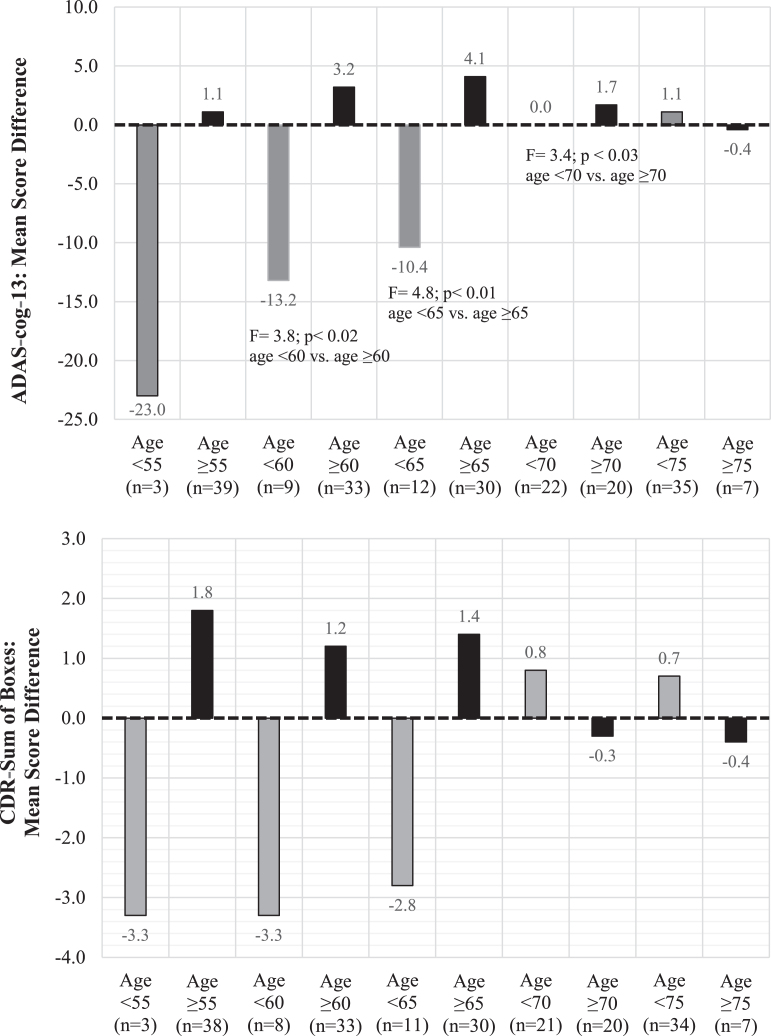
ADAS-cog-13 and CDR-SB mean score differences between DBS-f “on” and “off” groups from baseline to 12 months stratified by different age cutoffs^*^. ^*^Each bar represents the mean score difference between the DBS “on” versus DBS “off” participants in each age category. Positive mean score differences indicate that the DBS-f “on” group had less decline than the DBS-f “off” group whereas negative mean score differences indicate that the DBS-f “on” group had more decline.

Figure 2 displays mean observed score differences between the DBS-f “on” and “off” groups on the ADAS-cog-13 and CDR-SB scores stratified by 5-year age intervals. As shown by this sensitivity analysis, DBS-f “on” assigned participants < 65 years old did worse than DBS-f “off” participants on both the ADAS-cog-13 and CDR-SB, whereas DBS-f “on” participants≥65 years old did better than the “off” group.

Table 2 compares mean change scores from baseline to 12 months for the clinical metrics in the ITT population and the two *post-hoc* defined age cohorts. As shown, the DBS-f “on” group did significantly worse than the “off” group on all clinical measures in the < 65 age cohort. *Cohen*’*s d* effect size estimates (ES) favored DBS-f “off” versus “on” in the < 65 age cohort on all of the outcomes. The mean iADRS change score difference between the DBS-f “on” and “off” groups was 0.4 in the ITT population but increased to a 21.4-point difference favoring the DBS-f “off” group in the < 65 age cohort (ES = 1.41).

**Table 2:** Outcome score changes from baseline to 12 months stratified by age cohorts

	ITT population		Participants ≥65 years old		Participants < 65 years old	
	DBS-f “off”	DBS-f “on”	DBS-f “off”	DBS-f “on”	DBS-f “off”	DBS-f “on”
Enrolled (*n*)	21	21	15	15	6	6
**ADAS-cog-13**
Mean change ± SD	8.0±1.9	8.0±2.2	7.8±2.1	3.7±1.5	8.3±4.5	18.7±4.1
Difference ± SD		0.0±2.9		4.1±2.6		–10.3±6.1
*p*		ns		0.12		0.12
Cohen’s d (ES)		**0.00**		**0.58**		**–0.97**
**CDR-SB**
Mean change ± SD	2.4±0.4	2.7±0.7	3.5±0.9	2.1±0.5	0.5±0.3	3.4±0.8
Difference ± SD		–0.3±0.8		1.4±1.0		–2.9±0.8
*p*		ns		0.17		0.006
Cohen’s d (ES)		**0.09**		**0.52**		**–2.16**
**CDR-Global**
Mean change ± SD	0.4±0.1	0.4±0.1	0.5±0.1	0.4±0.1	0.1±0.1	0.6±0.2
Difference ± SD		0.0±0.1		0.1±0.2		–0.5±0.2
*p*		ns		0.38		0.02
Cohen’s d (ES)		**–0.11**		**0.32**		**–1.63**
**ADCS-ADL-23**
Mean change ± SD	–9.8±2.9	–9.5±1.5	–12.0±3.6	–7.9±1.8	–3.2±3.1	–13.3±2.3
Difference ± SD		0.3±3.2		4.1±4.0		10.1±3.8
*p*		ns		0.32		0.02
Cohen’s d (ES)		**–0.03**		**–0.37**		**1.63**
**iADRS**
Mean change ± SD	–15.7±4.0	–16.1±3.0	–17.3±5.9	–8.0±2.4	–8.4±7.0	–29.8±6.0
Difference ± SD		0.4±5.0		9.3±6.4		–21.4±9.2
*p*		ns		0.16		0.04
Cohen’s d (ES)		**0.02**		**–0.52**		**1.41**

Positive mean change scores indicate worsening for the ADAS-cog-13, CDR-SB, and CDR-Global scores, whereas negative mean change scores indicate worsening for the ADCS-ADL-23 and the iADRS. A positive mean difference between the assigned treatment groups indicates that the DBS-f “on” group had less decline than the “off” group over 12 months on that metric, whereas a negative mean difference between the groups indicates that the DBS “on” group has had more decline than the “off” group. Students’ *t* tests were used to calculate the *p* value. Positive Cohen’s d (effect size) favors DBS-f “on” group for ADAS-cog-13, CDR-SB, and CDR-Global; negative effect size favors DBS-f “on” group for ADCS-ADL-23 and iADRS.

In contrast, the DBS-f “on” group did substantially better than the DBS-f “off” group in the≥65 age cohort. The ES calculation favored DBS-f “on” in the≥65 age cohort on all of the clinical metrics. Although not statistically significant, the ES for the ADAS-cog-13 favoring the DBS-f “on” treatment group increased from 0.00 in the entire ITT population to 0.58 in the 30 participants≥65 years old, and the ES for the CDR-SB increased from 0.09 to 0.52.

In the≥65 age cohort, the mean iADRS change score increased to a 9.3-point difference favoring the DBS-f “on” group versus the DBS-f “off” group. The ES for the iADRS improved from +0.02 in the ITT population to –0.52 favoring DBS-f “on” in the≥65 age cohort.

**Fig. 3:**
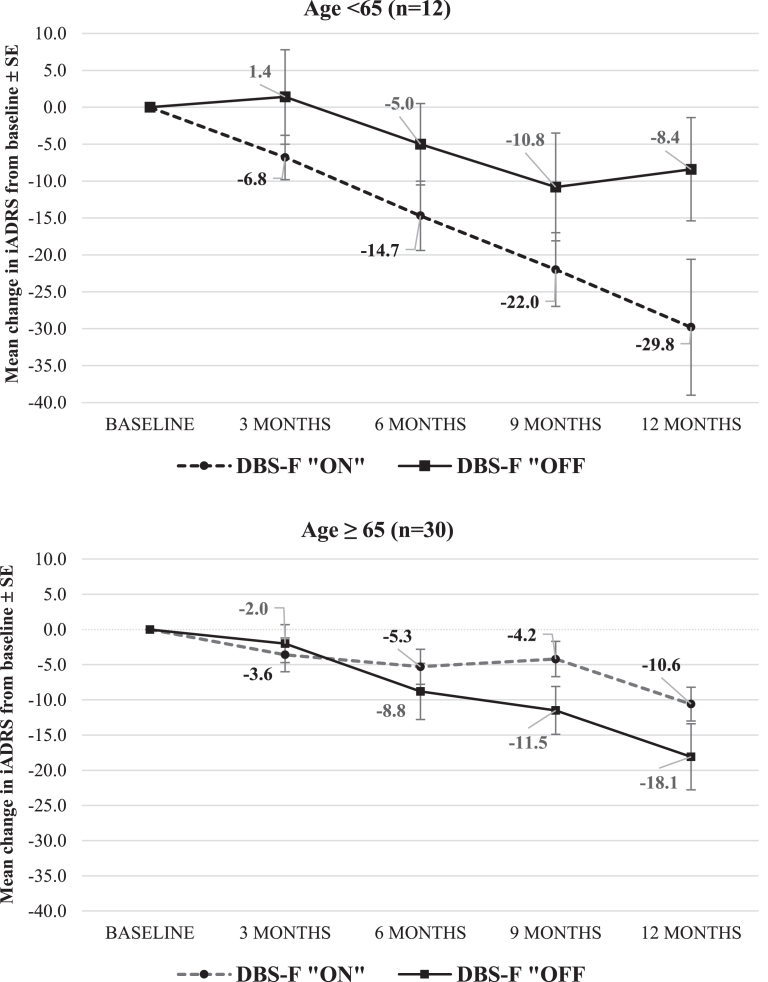
Change in iADRS from baseline to month 12 in ADvance 1 study. iADRS is the integrated Alzheimer’s Disease Rating Scale; negative mean change. scores indicate worsening from baseline.

Figure 3 displays the trajectory of the mean iADRS scores in the two age cohorts.

### Individual participant analyses

**Fig. 4:**
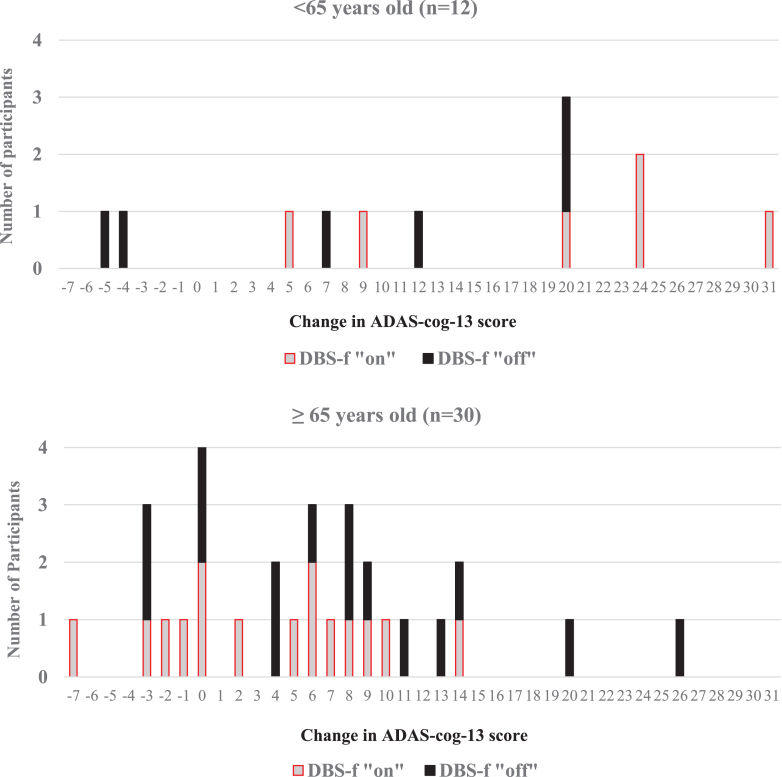
Distribution of ADAS-cog-13 score changes from baseline to 12 months. Negative ADAS-cog-13 change scores for each participant reflect improvement from baseline whereas positive change scores reflect progressive cognitive worsening from baseline.

Figure 4 displays individual ADAS-cog-13 score changes recorded between the baseline and 12 -month visits for each of the 42 enrolled participants. Notably, 8 participants had greater than a 20-point ADAS-cog-13 score worsening over 12 months. Six of the “outlier” participants were in the < 65 age cohort (representing 50%of the younger cohort) in contrast to only 2 of the 30 participants (6.7%) in the≥65 age cohort (χ^2^ = 7.82; df = 1; *p* = 0.005). The presence of 6 outliers in the < 65 age cohort may explain the marked differences observed between the “on” and “off” groups between the younger and older age cohorts and warrants further examination of the younger patient cohort.

**Fig. 5:**
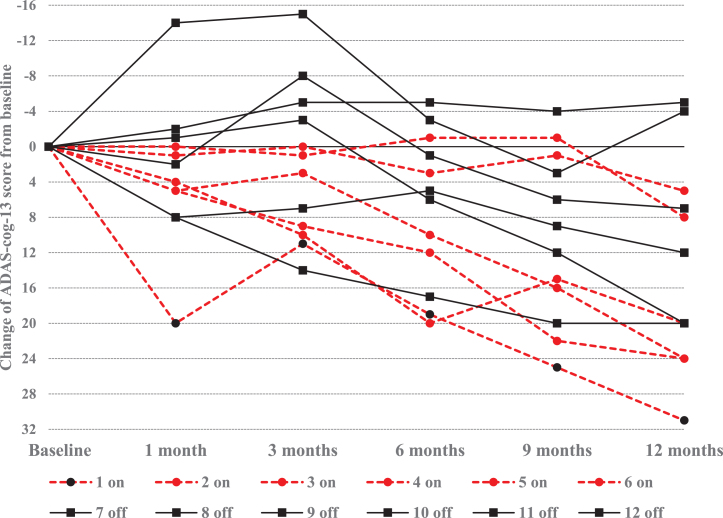
Trajectory of ADAS-cog-13 score changes in AD participants < 65 years old. Graphic reflects individual ADAS-cog-13 score changes from baseline in the DBS-f “on” and DBS-f “off” assigned participants < 65 years old; Negative change scores indicate improvement whereas positive scores indicate worsening from baseline.

Table 3 lists the available ADAS-cog-13 and CDR-SB scores for each of the 12 participants in the < 65 years age cohort at each study visit. The ADAS-cog-13 trajectories for the < 65 years age cohort are displayed in Fig. 5. Four of the 6 younger participants who had≥20-point score change (worsening) on the ADAS-cog-13 were randomly assigned to the DBS-f “on” group and the other 2 were assigned to the “off” group.

**Table 3:** Trajectory of ADAS-cog-13 and CDR-SB scores in AD participants < 65 years old

Patient	1 on	2 on	3 on	4 on	5 on	6 on	7 off	8 off	9 off	10 off	11 off	12 off
age	51	57	58	59	61	64	48	52	57	58	59	62
sex	m	f	f	f	m	f	m	f	f	f	m	m
		ADAS-cog-13 scores											
Baseline	24	34	27	28	25	30	29	27	30	20	20	30
1 month	44	38	32	28	26	35	15	35	29	22	18	38
3 months	35	44	30	29	25	39	14	41	27	12	15	37
6 months	43	54	37	27	28	42	26	44	36	21	15	35
9 months	49	49	43	27	26	52	32	47	42	26	16	39
12 months	55	54	51	36	30	54	25	47	50	27	15	42
*Δ* baseline-12 months	**31**	**20**	**24**	**8**	**5**	**24**	**–4**	**20**	**20**	**7**	**–5**	**12**
	CDR-SB scores											
Baseline	3.5	6.5	5	3	5	7	4.5	4.5	4.5	1.5	3	4
3 months	5	4.5	5	4	4.5	9	4	4.5	8	1	2.5	4
6 months	5.5	6.5	5	5	5.5	11	4.5	6	4	0.5	1	4.5
9 months	8	9	5	5	5.5	10	4.5	7	6	2	2.5	5
12 months	9	9		6	5.5	12	5	6	4.5	2	2.5	5
*Δ* baseline-12 months	**5.5**	**2.5**	0.0^*^	**3.0**	**0.5**	**5.0**	**0.5**	**1.5**	**0.0**	**0.5**	**–0.5**	**1.0**

The designation of “on” indicates that the patient was assigned to DBS-f stimulation treatment whereas “off” indicates that the patient was assigned to sham treatment after the surgical implant. *Δ* baseline-12 months reflects score change from baseline to 12 months where positive scores reflect cognitive and/or functional worsening. ^*^Last observation carried forward from CDR-SB assessment at 9 months.

As noted above, the ADAS-cog-13 and CDR-SB scores were correlated at baseline and 12-months (*r* = 0.459 and *r* = 0.676 respectively). Further, the magnitude of score changes over 12 months between the ADAS-cog-13 and CDR-SB were also correlated (*r* = 0.463 for the ITT population). Therefore, it is noteworthy that the sequential visit scores for participants 3 and 9 revealed marked progressive worsening on the ADAS-cog-13 score but no change at all on the CDR-SB during the course of the study (Table 3). The ADCS-ADL-23 and iADRS scores progressed similarly to the ADAS-cog-13 in both age cohorts since the composite score is based upon these instruments.

## DISCUSSION

We conducted a pilot study of deep brain stimulation targeting the fornix (DBS-f) in 42 participants with mild probable AD. Given the exploratory nature of the study, participants as young as 45 years old were eligible to participate provided they met study eligibility criteria. As a result, the study enrolled a disproportionately high number of participants who were < 65 years old (28.6%) relative to the < 4%that is reported in the AD population at large [16, 17]. The study achieved its primary objectives in demonstrating that DBS-f treatment in elderly adults with cognitive decline is both feasible and safe. However, none of the planned secondary clinical efficacy outcomes differentiated between the DBS-f “on” (stimulation) or “off” (sham) groups in the ITT population over the 12-months of double-blind treatment. As reported in this *post-hoc* analysis, age was a moderating factor that affected all study outcomes. The findings suggest that age was a proxy for the underlying heterogeneity often seen in younger AD participants.

In the cohort≥65 years old (*n* = 30), all of the clinical metrics and glucose metabolism measures favored DBS-f “on” treatment over the DBS-f “off” sham treatment after 12 months, although the difference did not reach statistical significance [6]. The study was not powered to show statistically significant differences between the treatment groups. However, the effect size (ES) calculations favored DBS-f “on” versus “off” in the age cohort≥65 years old and improved on other clinical metrics relative to the ITT population (Table 2). Conversely, the younger participants < 65 years old in the DBS-f “on” group did significantly worse than the “off” group on the CDR measures, ADCS-ADL-23, and iADRS.

We added the iADRS for this present *post-hoc* analysis. The EXPEDITION 3 study of solanezumab used the mean change score of the iADRS as the primary efficacy measure in an 80-week double-blind study that enrolled over 2000 participants with mild AD [22]. Over 80 weeks, the iADRS score worsened between 12–15 points from baseline in each group with a 1.7-point mean change score difference favoring solanezumab over placebo at the endpoint (*p* = 0.05). The iADRS was also the primary efficacy measure in the TRAILBLAZER-ALZ trial of donanemab that enrolled 257 AD participants [23]. After 76 weeks, the mean change score difference of the iADRS was 3.2 points favoring donanemab over placebo (*p* = 0.04). In this much smaller ADvance study (42 AD participants), the mean change score difference of the iADRS after 52 weeks was 9.3 points favoring DBS-f “on” versus “off” in the≥65 age cohort (ES = –0.52) but 21.4 points favoring the “off” group in the < 65 age cohort (ES = 1.41).

Eight participants in this study had marked worsening of the ADAS-cog-13 score over 12 months that exceeds the progression typically seen in mild AD [4, 21]. Six of these participants were < 65 years old (50%of the age < 65 cohort) in contrast to only 2 of the 30 participants (6.7%) in the older age cohort (*p* = 0.005). Four of the 6 younger rapidly declining participants were randomly assigned to DBS-f “on” (Table 3). It is possible that the DBS-f stimulation exacerbated the rapid cognitive decline in these younger participants, although the variability of some individual participant ADAS-cog-13 and CDR-SB responses as displayed in Table 3 suggest otherwise. It is more likely that older participants (≥65 years old) were less impaired than the younger AD participants who had different, more aggressive subtypes of AD than the older cohort or competing cognitive diseases that influenced their rapid decline. It is noteworthy that the pre-operative PET scans of the participants < 65 years old revealed significantly lower glucose metabolism than the older participants in both temporal and parietal areas (middle temporal gyrus, inferior parietal lobule, precuneus; –6 to –11%decrease; *p* < 0.05) [6].

The observed outcome differences noted between the younger and older AD participants may be related to genetic subtypes of AD that cause greater brain atrophy, metabolic deficits, and a more malignant course in younger participants [1–4, 32–34]. Three genes have been associated with early onset AD (amyloid precursor protein and the presenilins 1 and 2) and presenilin 1 has been identified in frontotemporal dementia, which lacks amyloid pathology [33]. Although we did not do genetic testing in this study, participants were excluded if it was known that there was a family history of familial autosomal dominant AD. The apolipoprotein E (*APOE*) E4 allele has also been identified as a risk factor for late onset AD [3]. Although all participants had typical AD hypometabolic patterns in their pre-operative PET scans, it is also possible that some of the younger participants did not have AD at all or had competing diseases that affected their clinical outcome and disease trajectories. Beyond genetic subtypes that affect the course of early onset AD, Schneider and colleagues [4] have also suggested that older participants with probable AD might have non-Alzheimer neuropathology (e.g., vascular disease, hippocampal sclerosis, TDP-43 proteinopathy) and/or decline more slowly than younger participants because of survival bias by which more rapidly declining individuals have progressed to a point where they are no longer eligible for the studies.

Several limitations related to this report must be noted. First, this was a *post-hoc* analysis of a defined sub-group within the ITT population and does not change the fact that the overall study did not reveal notable differences between the DBS-f “on” and “off” groups on any clinical metrics. We did not anticipate an over-representation of early onset AD when the protocol was written, and therefore did not specify age stratification in the initial statistical analysis plan. Second, ADvance was an exploratory phase II feasibility trial that intentionally allowed a wide age range and did not require ApoE or CSF biomarkers for eligibility. Although we confirmed that all enrolled patients had evidence of temporal-parietal hypometabolism prior to randomization, we cannot affirm that study participants had specific biomarkers consistent with probable AD. Third, the study sample was very small and not powered for statistical significance on any of the clinical metrics. Consequently, the *post-hoc* findings reported here must be interpreted with caution and require larger studies to address the clinical benefit that might be accrued by DBS-f treatment in mild AD.

These results highlight the importance of subject selection in clinical trials and the potential confounding effect that heterogeneous study populations can have on study outcomes. In this study, the recruitment of a disproportionately high number of younger AD participants adversely affected the outcome. In retrospect, it is not possible to determine whether other AD studies have failed because the candidate treatment intervention was ineffective and/or because the enrolled study population was too heterogeneous to detect a significant clinical effect (signal detection) in a sensitive (responsive) subtype. Patient recruitment is a challenge in all clinical trials and was a particular challenge in this study that required that the potential participant with mild AD consent to neurosurgery with the support and signed consent of a family member [35]. The recruitment challenges for this type of study were recently reported by Fontaine et al. [36] who noted that enrollment into a European DBS clinical trial for AD was extremely difficult. In that study, only 9 of 110 AD participants met study eligibility criteria and only one patient actually consented to participate in the neurosurgical procedure [36]. The screen failure rate of approximately 50%in the present study was consistent with most AD studies and, in that regard, was more successful than the European DBS study.

Despite several innovative strategies and more than 1000 AD clinical trials in the past two decades, no new treatments have been approved since 2003 [35, 37–39]. During this same time, investigators have differentiated clinical, genetic, and neuropathological subtypes of AD and recognized multiple competing diseases subsumed within the so-called AD population that might obscure the interpretation of clinical trial results [3, 5, 31–33, 39–49]. The lack of successful trials may be due, in part, to the heterogeneous populations of probable AD participants who were enrolled some of these studies. In a recent review, Ferreira and colleagues [5] emphasized that differentiation of the heterogeneity that exists within the AD population is critical for implementing precision medicine approaches and for ultimately developing successful treatments for AD. The findings from the ADvance study reinforce the importance of heterogeneity within AD and suggest that more restricted age limits, genotyping, and CSF biomarkers need to be part of the eligibility criteria for future AD trials.
